# A Brief Notice of “The Artesian Well and Saline Baths,” at St. Catherines, Canada West

**Published:** 1856-09

**Authors:** Theophilus Mack


// BUFFALO MEDICAL JOURNAL

AND

MONTHLY REVIEW.

VOL. 12. SEPTEMBER, 1856. NO. 4.

### ORIGINAL COMMUNICATIONS.

ART. I.—*A Brief Notice of "The Artesian Well and Saline Baths," at St. Catherines, Canada West.* By THEOPHILUS MACK, M. D.

During the last four years, strong efforts of a popular nature have been put forth to attract the attention of the public to a new watering-place at this town. These exertions have been so far crowned with a success highly encouraging to the proprietor; last season a crowd of visitors resorted hither, and went away fully satisfied, many of them to return again, and this summer the rooms at the large hotel and the boarding places in its vicinity, are now fully occupied; nearly all the accommodation that can be afforded is taken up at this date, viz., 22d of July.

Most of the parties arriving for valetudinary motives, appear to be quite unacquainted with the therapeutic properties of the spring, either in the form of an internal remedy, or as a bath, and go to work recklessly bathing and drinking without regard to diet or regimen, in a manner that proves sometimes quite disastrous; and some come, or are sent here, with a class of complaints to which the spa is wholly unsuited. Many complain that their medical attendants at home appear quite ignorant of the properties or even the existence of such a mineral water, and from this latter cause practitioners have discouraged persons from coming, who, nevertheless, have been peculiarly benefited by their disobedience.

With the view of affording information to the profession, from a responsible source, as to the class of patients likely to be improved by a sojourn here, I take the liberty of submitting in the pages of this Journal, the following resumé of the chief points connected with the spa, that may prove interesting or necessary, before recommending an excursion to a client.

My own experience of the qualities of the "St. Catherine's Well," extends over a period of about five years, and I can speak in the highest terms of the efficacy of the baths in chronic rheumatism, and during convalescence from the acute form of that disease. In engorgement, hypertrophy or inflammation of the neck or lower segment of the uterus, and chronic internal metritis, they are most valuable indeed. I may speak in nearly the same terms of their effects in all tumors of the organs of generation in females of a non-malignant character; the combination of muriate of lime, bromine and iodine existing in the water, will naturally lead to the conclusion that it must modify the growth of, or remove hypertrophic enlargement or soluble tumors, by increasing the activity of the absorbing vessels. In one case last season, an enlargement of one ovary, of two years' duration, entirely disappeared, and in two cases of chronic inflammation of the ovary the cure was complete.

In convalescence from all diseases of malarious origin and in malarious cachexy, the use of the warm bath is always most satisfactory. The sallow œdematous denizén of the western prairies, soon begins to show the restitution of vigorous capillary circulation—in many such cases a sickly pallor, after three weeks' bathing, gives place to the blush of health.

The activity of the absorbents excited by the bath, causes a rapid diminution in induration and enlargements of the liver and spleen, and I infer the same result must ensue in all *soft* abnormal growths. In a few cases of psoriasis and lepriasis they have conduced to the establishment of convalescence.

The effect of the *hot* bath is highly stimulating, causing throbbing of the carotid, cephalalgia, and often spasmodic contraction of the muscles, especially in the extremities, continuing for a long time and seldom followed by much sedative action; the same train of phenomena are generally observed in the *warm* bath, in a subdued degree; occasionally, however, a very marked depression ensues, but seldom resulting in any debilitating effect; many persons even bathe twice daily at the temperature of 98° to 100° Fahr., and apparently gain strength daily, although persisting in such a course, (on their own prescriptions) for weeks. The tepid bath is eminently calmative, and produces the specific action of the water gradually and effectually; it also proves the best adapted to the majority of the affections of the uterus, ovaries and mamma. The water as pumped from the well is at a remarkably low temperature, and affords a most refreshing bath, eminently grateful in summer. A most luxurious softness of the skin throughout the remainder of the day, always follows the bath in any form. This is to be ascribed to the hydrometric properties of the chloride of calcium, which maintains a moisture on the surface for several hours.

The cold plunge and shower baths are invigorating and tonic, and indicated in some forms of chlorosis and anæmia; the saline constituents promote reäction.

In 1853 I made a careful *qualitative* analysis of the spa, and upon reference to my chemical memorandum-book, I find the result to have been as follows:

Taste: intensely saline.
Odor: marine.
Reäction: neutral.
Specific gravity: 1036, at 60° Fahr.
Temperature, 60°; Temp. of Air, 76° Fahr.
Chloride of Sodium.
" of Calcium.
" of Magnesium.
Sulphate of Lime.
Iodide of Magnesium.
Bromide of "
Carbonate of Iron.
Alumina.
Free Carbonic Acid.
Silica.

The same summer a careful quantitative analysis was made by Mr. Croft, of the "Toronto University," which was published by Mr. Stephenson, and I now subjoin a copy:

*Analysis of Prof. Henry Croft.*

| | | | |
|---|---|---|---|
| Sulphate of Lime, - - - - - | | 2.1923 | 16.8368 |
| Chloride of Calcium, - - - - - | | 14.8544 | 114.0818 |
| Chloride of Magnesium, - - - - | | 3.3977 | 26.0944 |
| Iodide of do. - - - - - | | 0.0042 | 0.0322 |
| Bromide of do. - | a trace. | | |
| Chloride of Potassium, - - - - - | | 0.3555 | 2.7302 |
| Chloride of Sodium, - - - - | | 29.8034 | 228.8901 |
| Chloride of Ammonium, Silicic Acid, | a trace. | 50.6075 | 388.6655 |
| | Loss, | 1.0670 | |
| Toronto, July 25, 1853 | | 51.6745 | |

In chemical composition, the water, it will be seen, approximates closely to the Creuznach maters in Germany, the efficacy of which as a bath more especially, is fully acknowledged abroad, and whither a considerable number from Great Britain have annually resorted the past two years, the attention of the public having been directed to them, by the eulogy of their merits, from Dr. Locock, Prof. Simpson, and many other physicians of repute.

Administered internally the effect is of a diuretic and cathartic character, occasionally emeto-cathartic (when taken very liberally.) When deprived of its common salt by concentrating and priority of crystalization, it is the most certain diuretic I know of. In the gouty diathesis it has always proved highly beneficial. The analysis shows that these waters are calculated to favor elimination most powerfully, and in the scrofulous diathesis they are truly remedial. Tr. ferri, sesquichloridi and some of the ioduretted preparations may be taken with them, and the ol. morrhua appears often to agree better and rest easier on the stomach, when exhibited while the patient is taking the bath and drinking the *mother water.*

In the atonic dyspepsy attended with acidity, and foul eructations, so prevalent among city residents, and in sarcinæ ventriculi, chloride of calcium, has been greatly extolled by Dr. Leared, Physician to the "Royal Infirmary for Diseases of the Chest," and I can add the most favorable testimony to its action in the first named class of affections. In the form in which it exists here, as a natural production, the modus operandi is possibly by precipitation of the excess of sulphuric acid or soluble sulphates in the stomach, and the decomposition of other acids, resulting in the formation of new chloride and the evolution of free chlorine. The action of chloride of calcium as laid down in the systematic works upon materia medica, it is unnecessary for me here to dilate upon, as well as to vindicate the claims of that remedy for rescue from the unmerited neglect into which it has fallen. The therapeutic value derived from the presence of the iodine, bromine, and iron, from the fact that they exist in a very high proportion, will be at once recognized, giving to the spa a very composite character, which may, perhaps, like that of Kissingen, give it a claim to the title of corrective and roborant, or like those of Marienbad and other German waters.

With a lack of information respecting the power of mineral waters in curing disease, there exists also with many medical practitioners a scepticism carried to the extent of sneering incredulously at all spas, whether acquainted with their analysis and qualities or not, and of ascribing to the influence of change of scene, air, and amusement, *all* the effects of a visit to a watering-place. Now most of these identical men when consulted in any protracted disorder will prescribe alteratives, saline draughts, cathartics, "et id genus omne," all containing soda, lime, magnesia, iron, or iodine; why, therefore, should they affect to despise combination of the same ingredients in the all-pervading solvent-water, effected by the cunning chemistry of nature, aided by telluric heat and pressure. We all know that disease is discharged from the human body, whether spontaneously or under the agency of remedies, by means of some variety of secretion; either boils or eruptions, mucous or serous discharges, diuresis or deposits, salivation, bleeding from the hæmorrhoidal veins, desquamations, perspiration or suppuration; most indubitably, then, do mineral waters in the form of bath, or taken into the stomach, produce all the effects enumerated, and they only require judicious application to reap from them all these benefits.

One thing it is most important to impress upon all parties visiting this place for the purpose of health, viz., to consult their own medical attendant first, and having obtained an outline of the features and treatment of their case, to wait patiently upon their arrival here, and obtain the advice of a physician—not recklessly to commence bathing at any temperature, and drinking the water at all hours, indulging at the same time in every variety of regimen.

St. Catherines is very prettily situated along the banks of the Twelve Mile Creek, which is taken advantage of for the Welland Canal. Behind the town are a range of heights running from the Niagara Falls to the more lofty hills in rear of Hamilton. The country above the heights is beautifully undulating, and abounds in picturesque drives; about three miles from the town is the debouchement of the canal into Lake Ontario, and the basin at Port Dalhousie, a place of much pleasing scenery. The air is most salubrious, and the town has never been visited with any serious epidemic. I am now making some statistical observations upon the curative power of the waters, and when these shall have been matured, I shall be prepared at some distant future day, to show more scientifically what we are to expect from their use.

